# TUBA1C is a potential new prognostic biomarker and promotes bladder urothelial carcinoma progression by regulating the cell cycle

**DOI:** 10.1186/s12885-023-11209-2

**Published:** 2023-08-01

**Authors:** Yi Jiang, Chao Zhu, Haoxuan Huang, Gaomin Huang, Bin Fu, Xiaoqing Xi

**Affiliations:** 1 Department of Urology, The Second Affiliated Hospital of Nanchang University, Nanchang, China; 2 Department of Blood Transfusion, The First Affiliated Hospital of Nanchang University, Nanchang, China; 3 Department of Urology, The Third Affiliated Hospital of Nanchang University, Nanchang, China; 4 Department of Urology, The First Affiliated Hospital of Nanchang University, Nanchang, China

**Keywords:** BLCA, TUBA1C, Cell cycle, Tumor immunology, Prognosis

## Abstract

**Background:**

TUBA1C is an α-tubulin isoform involved in mitosis, and its dysregulation has been implicated in tumor progression. There is still no clear understanding of its role in bladder urothelial carcinoma (BLCA).

**Methods:**

This study examined the differential expression of TUBA1C and its prognostic significance in bladder cancer based on data from The Cancer Genome Atlas (TCGA) and Gene Expression Omnibus (GEO) and also assessed the correlation of TUBA1C expression level with immune cell infiltration and immune checkpoint gene expression levels and the half-inhibitory concentration (IC50) of different chemotherapeutic agents. Immunotherapy response was estimated using the Tumor Immune Dysfunction and Exclusion (TIDE) algorithm. We detected TUBA1C expression in BLCA cells using PCR and Western blotting. Functional assays, including CCK-8, colony formation, transwell, apoptosis and cell cycle assays, were also performed to assess the oncogenic role of TUBA1C in BLCA.

**Result:**

In three independent public cohorts, TUBA1C was significantly upregulated in bladder tumor tissues, and high TUBA1C expression in bladder cancer was associated with a poorer outcome than low expression. TUBA1C was an independent prognostic risk factor for bladder cancer, and numerous immune checkpoint genes and infiltrating immune cells were associated with TUBA1C. TIDE analysis revealed that TUBA1C showed great potential for predicting the immunotherapy response in bladder cancer patients. In addition, drug sensitivity analysis revealed that high TUBA1C expression indicated sensitivity to multiple chemotherapeutic agents. Functional assays revealed that silencing TUBA1C significantly inhibited the proliferation, migration and invasion of BLCA cells and induced apoptosis and cell cycle arrest.

**Conclusion:**

The overexpression of TUBA1C in bladder cancer predicts a poor prognosis and may also be a potential immunotherapeutic target. As a prognostic marker, TUBA1C influences tumor progression by regulating the cell cycle.

**Supplementary Information:**

The online version contains supplementary material available at 10.1186/s12885-023-11209-2.

## Introduction

A recent study revealed that bladder cancer has a poor prognosis worldwide, and there are over 550,000 new cases of the disease every year [1]. More than 95% of bladder cancers are epithelial cancers, most of which are transitional papillary carcinomas, and nearly 1/3 of bladder cancers consist of multiple tumor types. Cancer cells that grow into the bladder cavity without invading the bladder muscle tissue are called non-muscle-invasive bladder cancer (NMIBC). These tumors are superficial, represent the early stage and are the most common type of bladder cancer tumor. When cancer cells invade the muscle layer of the bladder, the disease is called muscle-invasive bladder cancer (MIBC) [2]. There is an approximate 90% 5-year survival rate (OS) for patients with NMIBC; approximately 15–20% of NMIBC cases progress to MIBC, and only 60–70% of MIBC patients survive for 5 years past diagnosis. Approximately 20% of newly diagnosed BLCA cases are MIBC, and approximately 50% have distant metastases [3]. Although surgery combined with chemotherapy and radiotherapy can prolong patient survival, the prognosis is still poor [4]. Hence, it is very important to actively explore new and effective biomarkers to reveal the molecular mechanism of tumor progression to further improve the diagnosis rate, treatment and prognosis evaluation of BLCA.

Microtubules are unbranched hollow reticular structures composed of tubulin fibrils. They are mainly include the α/β-tubulin heterodimer [5]. α-Tubulin is expressed in cancer or normal tissues and is related to a poor prognosis in various cancers [6]. A positive correlation exists between the expression of β-tubulin and malignant biological behavior in different tumors [7, 8]. Microtubules constitute intracellular network scaffolds, interact with various organelles, support and maintain cell morphology, and participate in processes such as cell division, cell motility, intracellular tissue and organelle transport and signal transduction [9]. Microtubules are essential for regulating cell division, and their dysregulation can lead to cancers such as lung, breast, cervical, gastric, and pancreatic cancers [7, 10, 11]. TUBA1C is a multifunctional cytoskeletal protein belonging to the α-tubulin family [12]. TUBA1C overexpression predicts a poor prognosis in hepatocellular carcinomas (HCCs) and promotes cell proliferation and migration [13]. The TUBA1C gene regulates the cell cycle and promotes pancreatic ductal adenocarcinoma cell invasion and migration [14]. In lung adenocarcinoma and low-grade glioma, overexpression of TUBA1C has been associated with a poor prognosis [12, 15]. This evidence suggests that TUBA1C is closely connected to tumor progression. However, no studies of the prognostic value and mechanism of TUBA1C in BLCA have been published.

Currently, tumor immunity is a hot topic in cancer research. Tumor immunity affects the immune system and inhibits the formation of the immune microenvironment through various mechanisms, thus preventing the occurrence of an effective antitumor immune response [16, 17]. Therefore, a comprehensive understanding of the immune infiltration status of cancer patients is particularly important for the selection of the correct individualized immunotherapy. BC is closely connected to tumor immunity; multiple immune cells and inflammatory biomarkers have been reported to be related to BLCA, and some trials have proven the therapeutic benefits of immunotherapy for BLCA patients [17, 18]. Thus, there is a need to identify more biomarkers that can predict the prognosis of immunotherapy.

Multiple public databases were used to analyze the differential expression of TUBA1C and its prognostic role in BLCA, identify likely oncogenic pathways in BLCA based on GO and KEGG analysis, and discuss the correlations of TUBA1C with tumor immunity and drug response. Additionally, we examined the functional mechanism of TUBA1C in bladder cancer to determine its prognostic significance.

## Materials and methods

### Data sources

We downloaded the TCGA TARGET GTEx (PANCAN, N = 19,131, G = 60,499) cancer dataset from the UCSC (https://xenabrowser.net/) database. We extracted TUBA1C gene expression data for each sample. A total of 34 cancer samples (as shown in Supplementary Table 1) were obtained after eliminating samples with fewer than three replicates. In addition, we downloaded the normalized expression matrix and survival data of GSE13507 (https://www.ncbi.nlm.nih.gov/geo/query/acc.cgi?acc=GSE13507, which includes 10 normal bladder tissues, 58 paracancerous tissues and 165 BC tissues) and GSE32894 (https://www.ncbi.nlm.nih.gov/geo/query/acc.cgi?acc=GSE32894, which includes 308 uroepithelial tumor tissues) to verify the expression of TUBA1C in BLCA and to assess its potential prognostic role, and the TIDE score was obtained from the TIDE database (http://tide.dfci.harvard.edu). Samples with incomplete clinical information were considered ineligible and were excluded from the study. After preprocessing the data, including probe annotation, normalization, and correction, we applied the limma package in R software to perform differential gene expression analysis to assess differences in TUBA1C expression between normal samples and tumor samples.

### Identification of factors related to OS in BLCA

Clinical data of patients providing 19 normal bladder tissues and 412 BC tissues were extracted from the TCGA database. Clinical data included age, sex, clinical stage and histological grade. Survival data, including OS, disease-specific survival (DSS), progression-free interval (PFI), and disease-free interval (DFI), were used to analyze the relationship between TUBA1C expression and prognosis in BLCA. Based on the median value of TUBA1C expression, samples were divided into high and low expression groups. Cox regression analysis was used to evaluate independent prognostic factors. The R packages “survminer” and “survival” were also used to visualize TUBA1C’s prognostic value. Subsequently, to verify the independent prognostic value of the variables, Kaplan‒Meier survival analysis and Cox regression analysis were performed on BLCA samples from the GSE13507 and GSE32894 datasets.

### Correlation between TUBA1C and immune cell infiltration in BLCA

TIMER (https://cistrome.shinyapps.io/timer/) was used to examine the correlation between TUBA1C and BLCA tumor-infiltrating immune cells. With the R packages “ggplot2”, “ggpubr” and “ggExtra”, we investigated the connection between TUBA1C and 22 types of infiltrating immune cells. Based on the median TUBA1C expression level, patients were classified into high and low expression groups, and immune cell infiltration levels were compared between these groups. The TIDE algorithm was used to predict the immunotherapy response and its potential association with TUBA1C expression level [19].

### GO and KEGG enrichment analyses

We used the R packages “enrichplot”, “ggplot2”, “circlize” and “org.Hs.e.g.db” for GO feature annotation. GSEA software (v 4.1.0, http://www.broad.mit.edu/gsea) was used to evaluate the association of TUBA1C with related signaling pathways. The top six pathways with the highest normalized enrichment scores are shown in the graph [20, 21].

### Correlation of TUBA1C with drug sensitivity

Median inhibitory concentrations (IC50) are important indicators of drug effectiveness or sensitivity. Accurate prediction of the response to chemotherapeutics in the Genomics of Drug Sensitivity in Cancer (GDSC) database [https://www.cancerrxgene.org/] in different TUBA1C expression groups was achieved by the R packages “pRRophetic”, “ggpubr”, and “ggplot2”.

### Cell culture

Human BLCA cell lines (T24, 5637, EJ and J82) and a normal bladder uroepithelial cell line (SV-HUC-1) were cultured (all obtained from the Chinese Academy of Sciences Cell Bank: Shanghai, China). 5637, J82 and EJ cells were cultured in RPMI 1640 (Gibco) containing 10% fetal bovine serum (FBS; HyClone). Dulbecco’s modified Eagle’s medium (DMEM; Gibco) containing 10% FBS was used for the cultivation of T24 cells, and SV-HUC-1 cells were cultivated in F-12 K medium containing 10% FBS. All cell lines tested negative for mycoplasma contamination prior to the experiments.

### siRNA transfection

The siRNA for TUBA1C was purchased from RiboBio (Guangzhou, China). The siRNA sequences were as follows: NC-siRNA (5’-UUCUCCGAACGUGUCACGUTT-3’), TUBA1C-siRNA #1 (5’-GCTTCAAGGTTGGCATTAA − 3’); TUBA1C- siRNA #2 (5’- GAGCAATACCACAGCTGTT − 3’). Lipofectamine 2000 was used to transfect siRNAs into T24 and EJ cells.

### Real-time quantitative PCR (RT‒qPCR) analysis

By using Invitrogen TRIzol reagent, qRT‒PCR was used to measure TUBA1C and β-actin expression levels in the above cell lines. The 2^-ΔΔCt^ method was used to quantify TUBA1C and β-actin mRNA expression. The primers used were as follows: TUBA1C: forward primer, 5’-GACCTCGTGTTGGACCGAAT-3’, reverse primer, 5’- CGAGGTGAACCCAGAACCAG − 3’; β-actin: forward primer: 5’-CCCGAGCCGTGTTTCCT-3’, reverse primer: 5’-GTCCCAGTTGGTGACGATGC-3’.

### CCK-8 assay

T24 and EJ cells (3.0 × 10^3^ cells/well) in the logarithmic growth phase after transfection were inoculated in 96-well plates. A mixture of 10 µl CCK-8 reagent and 90 µl culture medium was added to each well at 0, 1, 2, 3 and 4 days. Cells were incubated at 37 °C for 2 h. An enzyme marker was used to measure the OD at 450 nm after incubation.

### Colony formation assay

We inoculated 600 cells/well of transfected T24 and EJ cells into six-well plates and cultured them in complete medium for 1–2 weeks to form single-cell colonies. Colonies were counted after fixing the cells in 4% paraformaldehyde and staining with crystal violet solution.

### Transwell assay

T24 and EJ cells (5.0 × 10^3^ cells/well) in the logarithmic growth phase after transfection were inoculated in 200 µl of serum-free medium in the upper chamber, and 600 µl of complete medium containing 20% fetal bovine serum was added to the lower chamber. The cells were incubated for 24 h at 37 °C; then, the cells remaining in the upper chamber were removed, and those that passed through to the lower chamber were fixed in 4% paraformaldehyde and stained with crystal violet. The cells were observed under a microscope, and for photography and counting, three fields of view were randomly chosen for each sample.

### Cell cycle and apoptosis assays

After transfection, T24 and EJ cells were digested with trypsin, rinsed three times with cold PBS and resuspended as single cells, and the cells were treated accordingly according to the instruction manual for the Cell Cycle and Apoptosis Assay Kit (C1052; Beyotime Institute of Biotechnology, Shanghai, China). processing. Flow cytometry (BriCyte E6 system) was used to analyze the cell cycle and apoptosis processes, and the results were statistically analyzed using FlowJo 10 software.

### Western blot analysis

RIPA lysis buffer (Thermo Scientific) containing phosphatase and protease inhibitors was used to extract cellular proteins, and the BCA Protein Assay Kit (Thermo Fisher Scientific) was used to measure proteins, followed by protein blotting. Information on all primary antibodies used in this study is as follows: TUBA1C (ab222849, Abcam), β-Actin (#3700, Cell Signaling Technology), Bcl-2 (#15,071, Cell Signaling Technology), Bax (#41,162, Cell Signaling Technology), Cyclin B1 (#12,231, Cell Signaling Technology), CDK1 (10762-1-AP, Proteintech), P27 (25614-1-AP, Proteintech) and P21 (10355-1-AP, Proteintech).

### Statistical analysis

A variety of software programs, including R version 3.6.1, GSEA version 4.1.0, GraphPad Prism 9.0, and ImageJ version 10.1, were used in the analysis of the data. Student’s t test was applied for each group of experiments. P < 0.05 was considered to indicate statistical significance.

## Results

### Differential expression of TUBA1C across cancers and prediction of tumor progression and a poor prognosis in BLCA

**Fig. 1:**
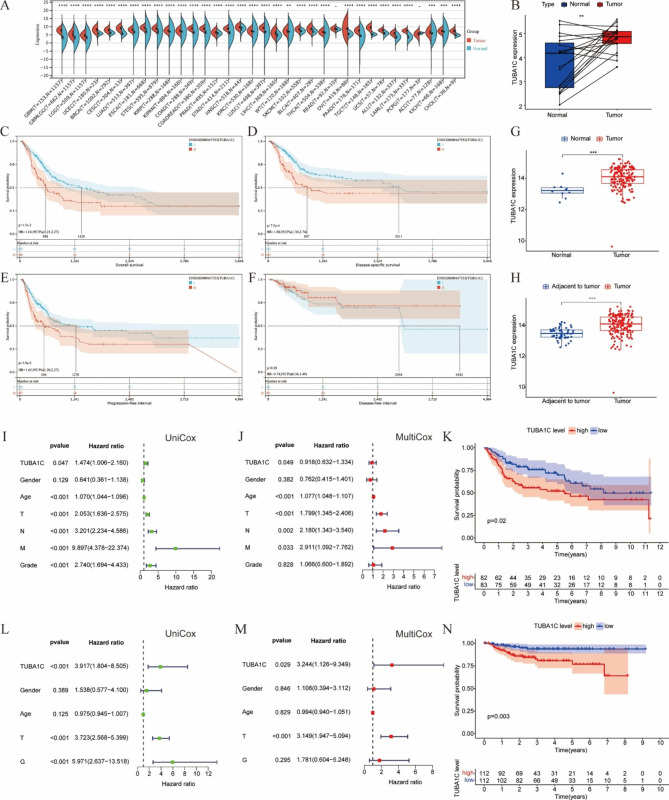
TUBA1C expression in normal and bladder cancer tissues. **(A)** TUBA1C gene expression in 34 tissues from the TCGA database; **(B)** Paired TUBA1C gene expression between normal and bladder cancer tissues from the TCGA cohort (*P* < 0.01). The expression of TUBA1C was significantly related to patient OS **(C)**, PFI **(D)** and DSS (P < 0.001) **(E)** in TCGA, only DFI (P = 0.39) **(F)**; TUBA1C expression was higher in bladder cancer tissues than in normal tissues **(G)** or adjacent normal tissues **(H)** from the GSE13507 cohort (*P* < 0.001). Associations between TUBA1C expression and overall survival and clinicopathological characteristics in the GSE13507 cohort according to Cox regression analysis **(I-J);** Kaplan‒Meier survival curve of overall survival for TUBA1C expression groups in the GSE13507 cohort (*P* = 0.02) **(K);** associations between TUBA1C expression and overall survival and clinicopathological characteristics in the GSE32894 cohort **(L-M)** according to Cox regression analysis; Kaplan‒Meier survival curve of overall survival for TUBA1C in the GSE32894 cohort (*P* = 0.003) **(N)**. **P < 0.01, ***P < 0.001, ****P < 0.0001

To compare TUBA1C expression levels in bladder tumors and normal tissues, we first analyzed the RNA-Seq data on TUBA1C expression in the TCGA pancancer dataset. We found that BLCA tissue samples had significantly higher mRNA levels of TUBA1C than normal bladder tissues. TUBA1C expression was also observed to be significantly upregulated in 31 other tumor tissues, as shown in Fig. 1A, and there was no significant difference in TUBA1C expression only in READ and pheochromocytoma and paraganglioma (PCPG).

Subsequently, TUBA1C expression levels in paired tumor tissue and adjacent tissue in the TCGA BLCA dataset were further evaluated and were found to be consistent with the previous results (Fig. 1B, P < 0.01). Based on TCGA data, Kaplan‒Meier analysis was used to assess the relationship between TUBA1C expression and patient survival/prognosis. Patients with high TUBA1C mRNA expression had a significantly poorer prognosis in terms of OS (p = 1.5e-3) (Fig. 1C), DSS (p = 7.5e-4) (Fig. 1D), and PFI (p = 1.9e-3) (Fig. 1E) but not DFI (p = 0.39) (Fig. 1F). The correlation between the mRNA expression of TUBA1C and prognosis in BLCA was not significant, indicating that the mRNA expression of TUBA1C in BLCA was correlated with prognosis. Some clinicopathological features were associated with poorer OS in a univariate Cox regression analysis (Table 1); these included advanced T stage (HR = 1.695, 95% CI = 1.1515–2.4949, p = 0.0074), advanced N stage (HR = 1.5312, 95% CI = 1.1646–2.0132, p = 0.0022), advanced overall tumor stage (HR = 1.7627, 95% CI = 1.2355–2.5147, p = 0.0017) and high expression of TUBA1C (HR = 1.0168, 95% CI = 1.0018–1.0320, P = 0.0278). TUBA1C was also independently associated with OS (HR = 1.6008, 95% CI = 1.0206–2.5108, P = 0.0404) in the multivariate analysis.

**Table 1:** Univariate analysis and multivariate analysis of the correlation of TUBA1C expression with OS among BLCA patients

BLCA	Univariate analysis			multivariate analysis		
	HR	95%CI	P value	HR	95%CI	P value
age	1.0277	0.9997–1.0565	0.0523			
gender	0.5881	0.3369–1.0263	0.0617			
stage	1.7627	1.2355–2.5147	0.0017	1.1944	0.5955–2.3956	0.6167
T	1.695	1.1515–2.4949	0.0074	1.437	0.8666–2.3827	0.1599
M	2.0869	0.7513–5.7966	0.158			
N	1.5312	1.1646–2.0132	0.0022	1.2488	0.7418–2.1024	0.4029
TUBA1C	1.0168	1.0018–1.0320	0.0278	1.6008	1.0206–2.5108	0.0404

Next, we validated these results in an external validation set using the same analytical approach. Differential expression of TUBA1C was assessed using data from GSE13507 (which includes information on 10 normal bladder tissues and 165 bladder cancer tissues), and TUBA1C expression was found to be elevated in BLCA (Fig. 1G, P < 0.001). Next, 58 adjacent normal bladder tissues and 165 bladder cancer tissues were compared. The results revealed that TUBA1C expression was similarly elevated in BLCA (Fig. 1H, P < 0.001). The GSE13507 and GSE32894 datasets showed that high TUBA1C expression was associated with higher mortality rate and shorter life expectancy (Fig. 1K, N). Cox regression analysis showed that high TUBA1C expression was independently and significantly associated with OS (Fig. 1I-J, L-M), and these results were consistent with previous TCGA-BLCA analysis results. Thus, TUBA1C is a major prognostic factor in BLCA patients that can be used for prognosis prediction.

### Immune cell infiltration in BLCA is associated with TUBA1C

**Fig. 2:**
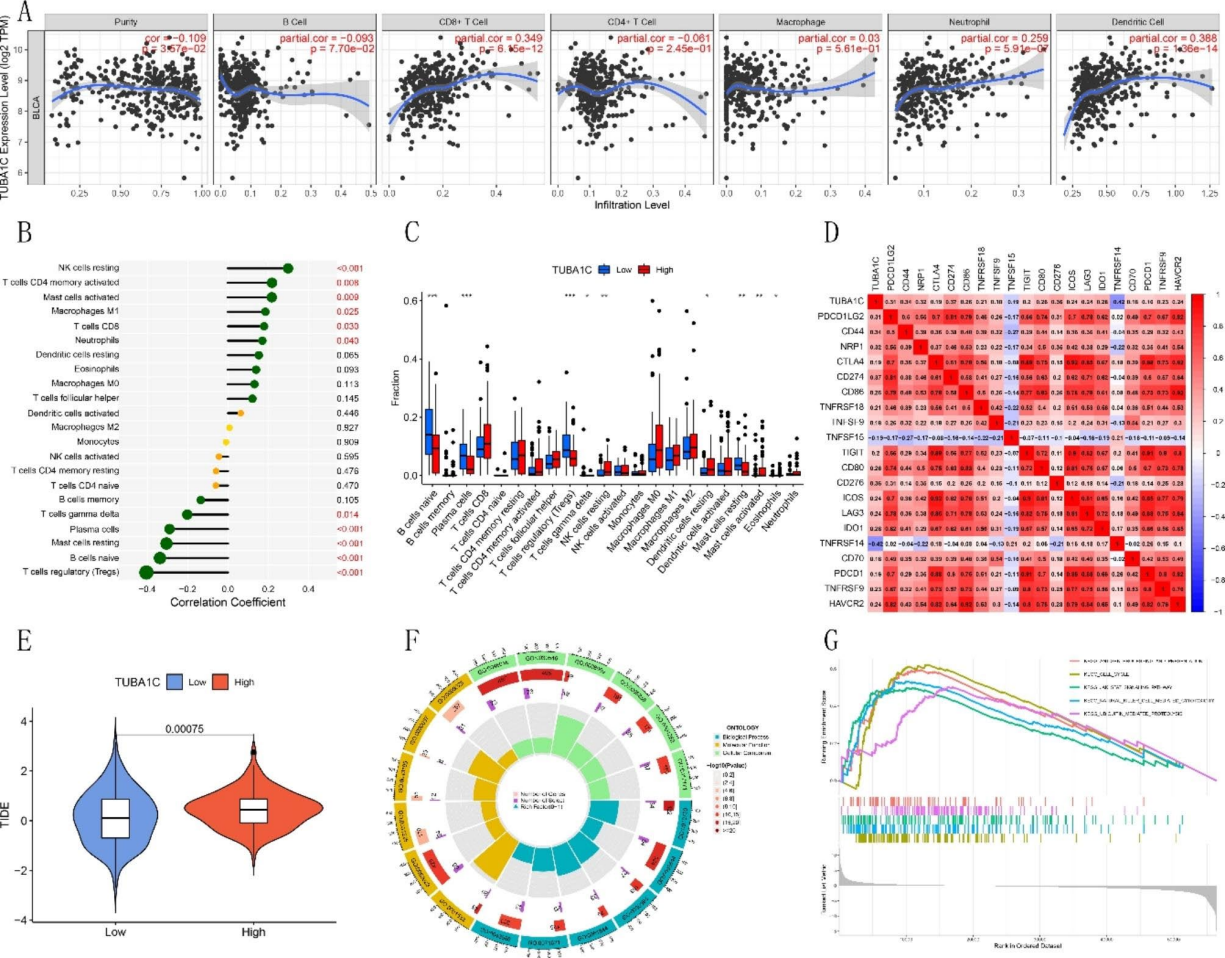
Correlation analysis between TUBA1C expression and the levels of infiltrating immune cells in BLCA. **(A)** TUBA1C expression level is related to the degree of immune infiltration in BLCA according to TIMER. **(B)** Lollipop diagram showing a correlation between TUBA1C expression and the levels of 22 tumor-infiltrating immune cells. **(C)** Proportions of 22 types of immune cells in different TUBA1C expression groups in the BLCA cohort. **(D)** A heatmap of the correlation matrix of immune cell proportions. The red color represents a positive correlation, and the blue color represents a negative correlation. **(E)** TIDE scores of the low and high TUBA1C groups of BLCA patients from the TIDE database. **(F)** Circle plot for GO functional enrichment analysis. **(G)** Enrichment of KEGG pathways. *P < 0.05, **P < 0.01, ***P < 0.001

The TIMER database was used to analyze the relationship between infiltrating immune cells and TUBA1C, and the analysis revealed that TUBA1C was significantly associated with the infiltration levels of B cells, CD8 + T cells, CD4 + T cells, macrophages, neutrophils, and dendritic cells (Fig. 2A). In a study involving 22 types of tumor-infiltrating immune cells, the CIBERSORT algorithm revealed a positive correlation between TUBA1C expression and the infiltration levels of resting natural killer (NK) cells, neutrophils, CD8 + T cells, M1 macrophages, activated mast cells, and activated memory CD4 + T cells. A negative correlation was found between the expression level of TUBA1C and the proportions of regulatory T cells (Tregs), naive B cells, mast cells, plasma cells, and gamma delta T cells. Resting NK cells had the strongest positive correlation (COR = 0.3), while Tregs showed the strongest negative correlation (COR = -0.4) with TUBA1C expression (Fig. 2B). The analysis also showed significant differences in the infiltration levels of naive B cells, plasma cells, regulatory T cells (Tregs), gamma delta T cells, resting NK cells, resting dendritic cells, resting mast cells, activated mast cells, and eosinophils between the TUBA1C expression groups (Fig. 2C, p < 0.05). Since TUBA1C is associated with immune infiltration, we further investigated the association between TUBA1C and common immune checkpoint genes. Significant associations between TUBA1C and 20 immune checkpoint genes were found in BLCA (Fig. 2D). Moreover, we observed a higher TIDE score in high expression group than in the low expression group (Fig. 2E), suggesting that immunotherapy is less effective in the high expression group. According to our findings, TUBA1C is involved in tumor immune infiltration in BLCA.

### Enrichment analysis

To further clarify the potential mechanism of TUBA1C in BLCA, enrichment analysis was performed. A total of 333 significantly enriched GO terms were found through GO functional annotation analysis. In the cellular component category, 73 terms, such as receptor ligand activity (GO: 0048018) and signaling receptor activator activity (GO: 0030546), were enriched; in the molecular function category, 60 terms, including collagen-containing extracellular matrix (GO: 0062023), were significantly enriched. In the biological process category, 54 terms, including epidermal development (GO: 0008544), were significantly enriched (Fig. 2F). Signaling pathways related to TUBA1C in BLCA were analyzed by GSEA (Fig. 2G). The top six pathways with the highest standardized enrichment scores in the high BLCA expression group were antigen processing and presentation, the cell cycle, apoptosis, natural killer cell-mediated cytotoxicity, bladder cancer, pathways in cancer and ubiquitin-mediated proteolysis. These results suggest an association between TUBA1C and tumor immune invasion/cell cycle in BLCA.

### Correlation of TUBA1C expression with drug sensitivity

**Fig. 3:**
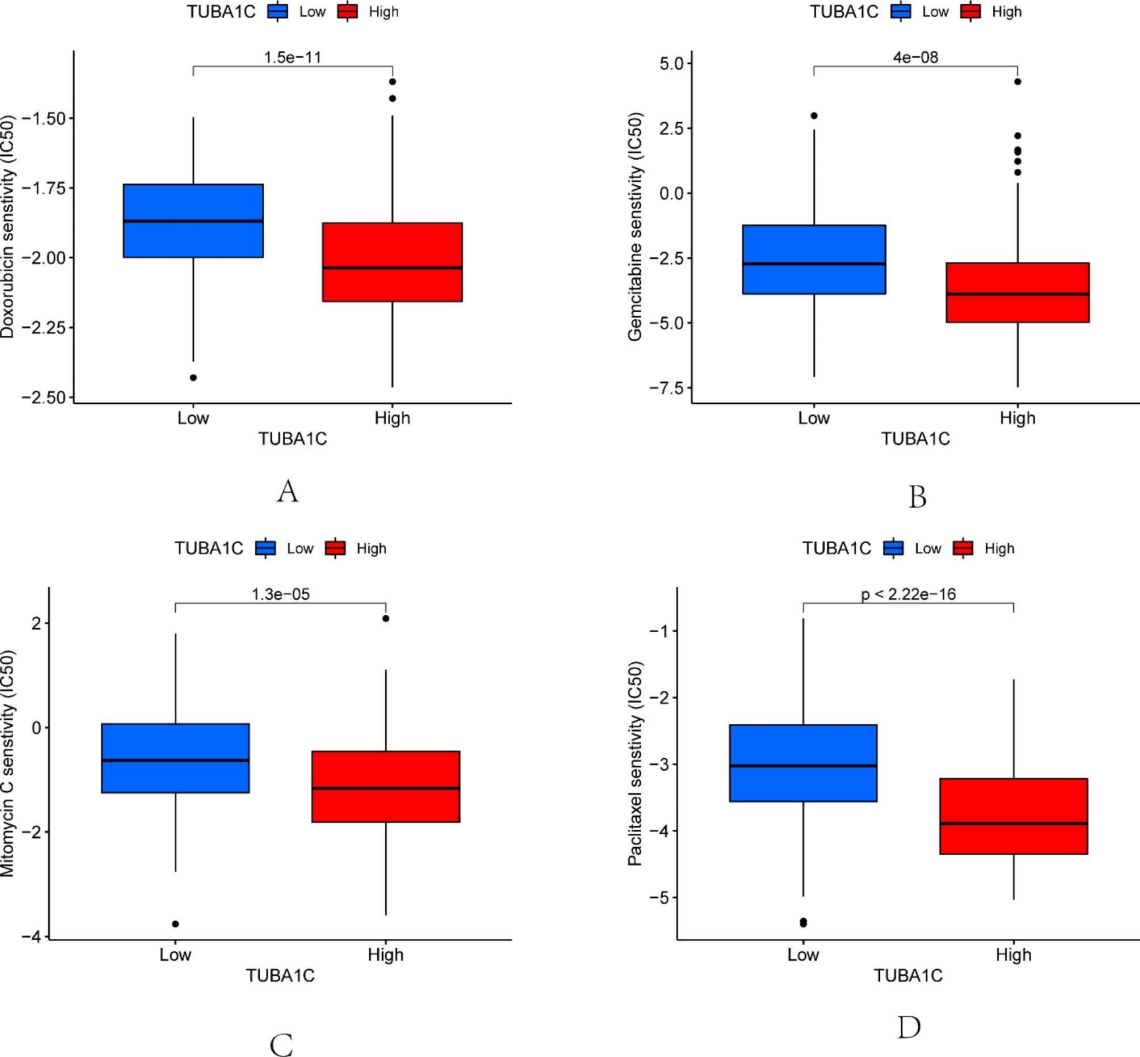
Distribution of IC50 scores of targeted drugs in different TUBA1C expression groups. **(A)** Doxorubicin, **(B)** Gemcitabine, **(C)** Mitomycin C, **(D)** Paclitaxel

We evaluated the relationship between sensitivity to chemotherapy drugs commonly used in BLCA and TUBA1C expression; mitomycin, doxorubicin, gemcitabine, and paclitaxel IC50 values were negatively correlated with TUBA1C expression (Fig. 3). These results suggest that TUBA1C expression may be a predictor of BLCA chemotherapy drug sensitivity.

### TUBA1C is upregulated in BLCA

**Fig. 4:**
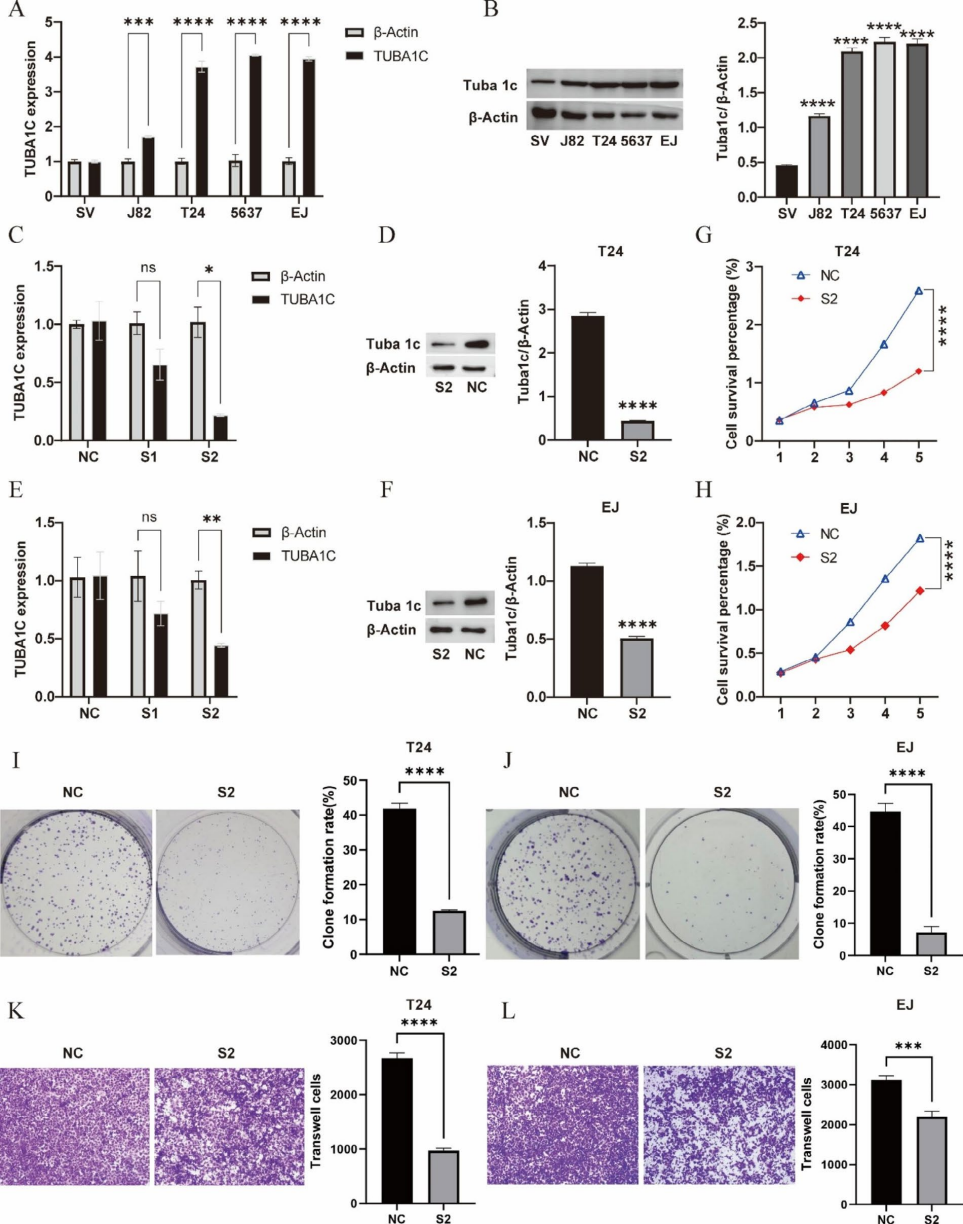
TUBA1C is differentially expressed in bladder cancer and normal cells, and silencing of TUBA1C expression inhibits the malignant progression of bladder cancer cells. qRT‒PCR **(A)** and WB analysis **(B)** of TUBA1C expression in bladder cancer cell lines; qPCR and WB analysis were used to confirm the efficiencies of TUBA1C overexpression and knockdown in T24 and EJ cells **(C-F)**; CCK-8 assay showing cell growth after knockdown of TUBA1C in T24 **(G)** and EJ cells **(H)**; Effect of knockdown of TUBA1C on the colony-forming ability of T24 **(I)** and EJ cells **(J)**; Transwell assay to detect the invasive ability of bladder cancer cells after silencing TUBA1C expression **(K-L)**. *P < 0.05, **P < 0.01, ***P < 0.001, ****P < 0.0001

To assess the role of TUBA1C in BLCA, we measured TUBA1C expression in BLCA cells by qRT‒PCR. The expression of TUBA1C mRNA in BLCA cell lines (T24, J82, 5637 and EJ) was significantly higher than that in normal bladder uroepithelial cells (SV-HUC-1) (Fig. 4A). Western blot analysis revealed a higher protein expression level of TUBA1C in T24, J82, 5637 and EJ cell lines than in SV cell lines (Fig. 4B). This finding indicates that TUBA1C is upregulated in BLCA cells. Considering that T24 and EJ cells express the highest levels of TUBA1C among the assessed cell lines, these two cell lines have strong tumorigenic potential in vivo and in vitro.

### Silencing TUBA1C inhibits the proliferation, migration and invasion of BLCA cells

We transfected T24 and EJ cells with siRNA-NC, TUBA1C#1 and TUBA1C#2 siRNAs and then verified the transfection efficiency by qRT‒PCR and Western blot analysis. A significant reduction in the expression of TUBA1C was observed after EJ and T24 cells were transfected with TUBA1C #2 siRNA (Fig. 4C-F), so TUBA1C #2 siRNA was selected for subsequent experiments and named S2-TUBA1C.

According to the CCK-8 results, T24 and EJ cell proliferation was significantly diminished at 2 d, 3 d and 4 d after transfection compared to that in the siRNA-NC group (Fig. 4G-H, P < 0.001). TUBA1C knockdown significantly inhibited colony formation in T24 and EJ cells (Fig. 4I-J, P < 0.001). Transwell assays also showed that downregulating TUBA1C significantly suppressed the invasion of T24 and EJ cells (Fig. 4K-L, P < 0.001). The results suggest that TUBA1C silencing suppressed EJ and T24 proliferation, migration, and invasion.

### TUBA1C regulates BLCA cell cycle progression and promotes apoptosis

**Fig. 5:**
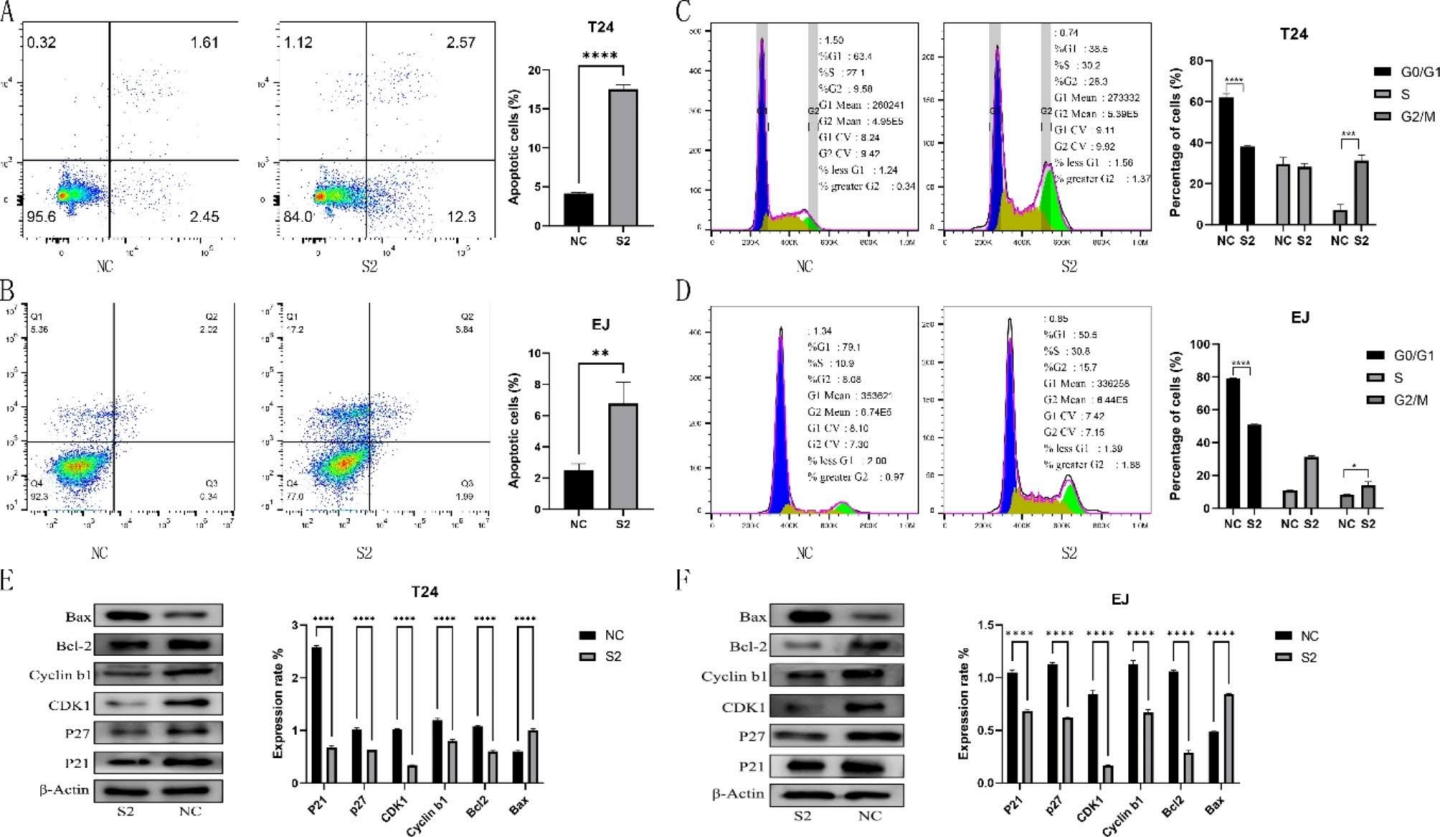
Effect of silencing TUBA1C expression on the cell cycle and apoptosis. **(A-B)** Flow cytometry apoptosis assay showing apoptosis in bladder cancer cells after knockdown of TUBA1C. **(C-D)** Flow cytometry cell cycle assay showing that knockdown of TUBA1C increased the fraction of G2 phase cells. **(E-F)** Western blotting was used to detect the expression of key apoptotic molecules and cell cycle-related proteins after knockdown of TUBA1C expression. (Although the spots were cut before hybridization with the antibody, interesting results were obtained after hybridization) *P < 0.05, **P < 0.01, ***P < 0.001, ****P < 0.0001

Based on the GSEA results, TUBA1C plays a role in the BLCA cell cycle and apoptosis. Therefore, we validated this idea experimentally. The results of flow cytometry showed that in T24 and EJ cells, the S2-TUBA1C group exhibited significantly higher levels of early, late, and total apoptosis than the siRNA-NC group (Fig. 5A-B, P < 0.001); TUBA1C downregulation significantly increased the percentage of G2 phase cells while decreasing the percentage of G1 phase cells (Fig. 5C-D, P < 0.001). In addition, the expression levels of cell cycle- and apoptosis-related proteins were measured by WB analysis. The group with downregulated TUBA1C had lower expression of Bcl2 expression and higher expression of Bax, which are apoptotic proteins; Cyclin B1, CDK1, P27, and P21 were also downregulated (Fig. 5E-F, P < 0.001). These data suggest that silencing TUBA1C induces BLCA cell arrest in the G2/M phase and promotes apoptosis.

## Discussion

Microtubules and tubulin are core factors that maintain cell homeostasis and carry out the cellular stress response; they influence protein signaling networks through molecular and organelle transport and act as scaffolds for protein–protein interactions involved in key biological functions, including cell division, cell movement, and the transport of intracellular factors and organelles [9, 22]. During cell division, for example, the microtubule network is usually assembled into a “mitotic spindle”, which is responsible for the separation of sister cells through recombination, depolymerization and reaggregation [23]. α/β-Tubulin heterodimers fuse into microtubules, which are essential for cell division and growth [24]. Multiple tumor types, such as breast, colon, prostate, liver, brain, bile duct, and pancreatic cancer, have been linked to microtubule regulation [22]. TUBA1C is involved in mitosis [12], and studies have reported that TUBA1C overexpression significantly affects the growth and progression of tumor cells [25]. However, the prognostic value and mechanism of TUBA1C in BC have not been studied.

Using GEO and TCGA datasets, we investigated the differential expression of TUBA1C in patients with BLCA. Analysis of three independent public cohorts revealed that bladder tumor tissues have higher TUBA1C expression than adjacent normal tissues; univariate/multivariate Cox analysis revealed that high TUBA1C expression was associated with higher mortality rates and shorter survival times. In addition, TUBA1C was found to be mainly associated with secreted biological functions by GO and KEGG pathway analysis of the TCGA database; genes coexpressed with TUBA1C were mainly enriched with pathways such as the cell cycle and apoptosis. There is evidence to suggest that TUBA1C can be used as a biomarker for BLCA.

In recent years, Zhu H et al. [15] showed that TUBA1C may promote the progression of low-grade glioma by regulating tumor immunity in the TME. Bian T et al. [12] discovered that immune invasion can modulate TUBA1C expression in LUAD, and the tumor immune microenvironment of LUAD was found to be regulated by TUBA1C. Therefore, we further explored whether TUBA1C is related to tumor immunity in BLCA. This study demonstrated a significant correlation between TUBA1C and the infiltration of CD8 + T cells, macrophages, neutrophils, and dendritic cells but not with B cells and CD4 + cells. TUBA1C was found to be closely related to 11 types of infiltrating immune cells, among which resting NK cells had the strongest positive correlation (COR = 0.3) and Tregs showed the strongest negative correlation (COR = -0.4). Among immune cells, Tregs and naive B cells had the highest correlation with TUBA1C expression levels. As intrinsic lymphocytes, NK cells play a crucial role in immunosurveillance and antitumor immunity. They play a role in inhibiting tumor growth and in regulating immune responses, and the initiation of the intrinsic immune response is regulated by the coordination and balance of inhibitory and activating receptors on their surface, both of which are potential targets for tumor immunotherapy [26, 27]. Regulatory T cells (Tregs) are essential for the response to tumor immunotherapy, as they play dual roles. On the one hand, Tregs contribute to the maintenance of the body’s autoimmune tolerance and minimize damage related to an excessive immune response, while on the other hand, Tregs can help cancer cells evade the body’s immune surveillance, which can facilitate tumor progression and metastasis. In the presence of Tregs, tumors are established and progress faster than they do in the absence of Tregs [28]. In addition, we further investigated the correlation between TUBA1C and common immune checkpoint genes. TIGIT, CTLA4, CD274, HAVCR2, LAG3, PDCD1, CD44, NRP1, CD276 and PDCD1LG2 are positively correlated with TUBA1C, and they all regulate the response to immune checkpoint blockade [29]. In this study, it was shown that the tumor microenvironment and immune response may be modified by TUBA1C, which may inhibit or promote cancer progression. Next, we further evaluated the relationship of TUBA1C expression with sensitivity to commonly used chemotherapeutic agents in BLCA; the results indicated that TUBA1C expression was negatively correlated with the IC50 values of doxorubicin, gemcitabine, paclitaxel and mitomycin C, indicating that TUBA1C expression is related to sensitivity to the above chemotherapy drugs. These results suggest that TUBA1C may be a predictor of BLCA chemotherapy drug sensitivity.

It is a major cause of cancer-related death and an indicator of disease progression reflected by tumor cell invasion. TUBA1C has been identified as a key gene that promotes tumorigenesis and is a potential new cancer target [30], and according to the literature, silencing TUBA1C inhibits pancreatic ductal adenocarcinoma cell proliferation, migration, and invasion [14]. The silencing of TUBA1C decreased cell proliferation and migration rates in hepatocellular carcinoma [13], and in NSCLC tissues, according to Yang J et al. [31], the expression of TUBA1C was upregulated, and silencing TUBA1C significantly inhibited cell proliferation and accelerated apoptosis. TUBA1C has also been shown to promote aerobic glycolysis by upregulating YAP expression to promote aerobic glycolysis and enhance lactate metabolism, glucose consumption, and cell growth, migration, and invasion, thereby promoting tumor progression in BRCA [32]. In our study, TUBA1C was significantly upregulated in BLCA, and its potential role in BLCA was revealed by silencing TUBA1C, which significantly suppressed BLCA cell migration and invasion, confirming its role in tumor progression.

There is increasing evidence that TUBA1C regulates cell cycle progression in various cancer types, and TCGA-based KEGG enrichment analysis revealed that TUBA1C may promote tumor progression in HCC and LUAD through cell cycle signaling pathways [12, 13]. With further studies, Yang J et al. [31] revealed that silencing TUBA1C decreased cyclin B1 expression and significantly promoted apoptosis in NSCLC cells. Studies in PDAC showed that silencing TUBA1C induced cell cycle arrest in PDAC cells at the G0/G1 phase, resulting in reduced expression of cell cycle-related proteins (cyclins D1 and E1 as well as CDKs 2, 4, and 6) [14]. Gui S et al. found that knockdown of TUBA1C induced a block in the G2/M phase and that the cell cycle-related proteins cyclin B1 and CDK1 were significantly reduced in glioma cells [33]. We found that silencing TUBA1C in T24 and EJ cells significantly increased the proportion of cells in G2/M phase and increased apoptosis, and Western blot analysis also revealed alterations in both cycle-related and apoptotic proteins. These data further suggest that silencing TUBA1C induces BLCA cell arrest in the G2/M phase and promotes apoptosis.

We investigated for the first time the prognostic value of TUBA1C and the correlation of TUBA1C expression with immune infiltration in BLCA. Our study demonstrates the expression of TUBA1C in BLCA and its prognostic value. TUBA1C may improve the effectiveness of immunotherapy by modulating immune infiltration and may be a predictor of immunotherapy and chemotherapy drug sensitivity. TUBA1C expression has been validated in the TCGA and GEO databases, as well as through in vivo experiments. TUBA1C significantly inhibits cellular proliferation, migration and invasion, induces cell cycle arrest and promotes apoptosis. Thus, TUBA1C may serve as a therapeutic target for BLCA based on its role as an oncogene. However, there are limitations to this study. Many clinical samples are required to verify its immune-related function, so the study needs to be replicated in other clinical samples. In the future, we will conduct more in vitro and/or in vivo experiments to explore the detailed mechanism of TUBA1C-mediated BLCA tumorigenesis.

## Electronic supplementary material

Below is the link to the electronic supplementary material.

Supplementary Material 1

Supplementary Material 2

## Data Availability

All data are available via the corresponding author. The datasets analysed during the current study are available in the TCGA database (https://xenabrowser.net/) and GEO database of GSE13507 (https://www.ncbi.nlm.nih.gov/geo/query/acc.cgi?acc=GSE13507) and GSE32894 (https://www.ncbi.nlm.nih.gov/geo/query/acc.cgi?acc=GSE32894).
