# Supplementary material for: TUBA1C is a potential new prognostic biomarker and promotes bladder urothelial carcinoma progression by regulating the cell cycle
Source: BMC Cancer. 2023 Aug 1;23:716. doi: 10.1186/s12885-023-11209-2 (PMC10391756; doi:10.1186/s12885-023-11209-2)
Supplement: Supplementary file 2 — Supplementary Material 2 [file 12885_2023_11209_MOESM2_ESM.doc]

Supplementary Table 1. 34 types of human cancers employed in our research.

Abbreviation Full name

ACC Adrenocortical carcinoma

BLCA Bladder urothelial carcinoma

BRCA Breast invasive carcinoma

CESC Cervical squamous cell carcinoma and endocervical adenocarcinoma

CHOL Cholangiocarcinoma

COAD Colon adenocarcinoma

COADREAD Colon adenocarcinoma/Rectum adenocarcinoma Esophageal carcinoma

ESCA Esophageal carcinoma

GBM Glioblastoma multiforme

GBMLGG Glioma

HNSC Head and neck squamous cell carcinoma

KICH Kidney chromophobe

KIPAN Pan-kidney cohort (KICH+KIRC+KIRP)

KIRC Kidney renal clear cell carcinoma

KIRP Kidney renal papillary cell carcinoma

LAML Acute myeloid leukemia

LGG Brain lower grade glioma

LIHC Liver hepatocellular carcinoma

LUAD Lung adenocarcinoma

LUSC Lung squamous cell carcinoma

OV Ovarian serous cystadenocarcinoma

PAAD Pancreatic adenocarcinoma

PCPG Pheochromocytoma and paraganglioma

PRAD Prostate adenocarcinoma

READ Rectum adenocarcinoma

SKCM Skin cutaneous melanoma

STAD Stomach adenocarcinoma

STES Stomach and Esophageal carcinoma

TGCT Testicular germ cell tumors

THCA Thyroid carcinoma

UCEC Uterine corpus endometrial carcinoma

UCS Uterine carcinosarcoma

ALL Acute Lymphoblastic Leukemia

WT High-Risk Wilms Tumor
