# Supplementary material for: TUBA1C is a potential new prognostic biomarker and promotes bladder urothelial carcinoma progression by regulating the cell cycle
Source: BMC Cancer. 2023 Aug 1;23:716. doi: 10.1186/s12885-023-11209-2 (PMC10391756; doi:10.1186/s12885-023-11209-2)
Supplement: Supplementary file 1 — Supplementary Material 1 [file 12885_2023_11209_MOESM1_ESM.docx]

β-Actin for Figure 4B

Tuba1c for Figure 4B

β-Actin for Figure 4D

Tuba1c for Figure 4D

β-Actin for Figure 4F

Tuba1c for Figure 4F

β-Actin for Figure 5E

Bax for Figure 5E

Bcl-2 for Figure 5E

CDK1 for Figure 5E

Cyclin b1 for Figure 5E

P21 for Figure 5E

P27 for Figure 5E

β-Actin for Figure 5F

Bax for Figure 5F

Bcl-2 for Figure 5F

CDK1 for Figure 5F

Cyclin b1 for Figure 5F

P21 for Figure 5F

P27 for Figure 5F
